# Abnormal dynamic functional network connectivity in patients with early-onset bipolar disorder

**DOI:** 10.3389/fpsyt.2023.1169488

**Published:** 2023-06-28

**Authors:** Ziyi Hu, Chun Zhou, Laichang He

**Affiliations:** Department of Radiology, First Affiliated Hospital of Nanchang University, Nanchang, China

**Keywords:** early-onset bipolar disorder, magnetic resonance imaging, dynamic functional network connectivity, brain imaging, brain functional network

## Abstract

**Objective:**

To explore the changes in dynamic functional brain network connectivity (dFNC) in patients with early-onset bipolar disorder (BD).

**Methods:**

Resting-state functional magnetic resonance imaging (rs-fMRI) data were collected from 39 patients with early-onset BD and 22 healthy controls (HCs). Four repeated and stable dFNC states were characterised by independent component analysis (ICA), sliding time windows and k-means clustering, and three dFNC temporal metrics (fraction of time, mean dwell time and number of transitions) were obtained. The dFNC temporal metrics and the differences in dFNC between the two groups in different states were evaluated, and the correlations between the differential dFNC metrics and neuropsychological scores were analysed.

**Results:**

The dFNC analysis showed four connected patterns in all subjects. Compared with the HCs, the dFNC patterns of early-onset BD were significantly altered in all four states, mainly involving impaired cognitive and perceptual networks. In addition, early-onset BD patients had a decreased fraction of time and mean dwell time in state 2 and an increased mean dwell time in state 3 (*p* < 0.05). The mean dwell time in state 3 of BD showed a positive correlation trend with the HAMA score (r = 0.4049, *p* = 0.0237 × 3 > 0.05 after Bonferroni correction).

**Conclusion:**

Patients with early-onset BD had abnormal dynamic properties of brain functional network connectivity, suggesting that their dFNC was unstable, mainly manifesting as impaired coordination between cognitive and perceptual networks. This study provided a new imaging basis for the neuropathological study of emotional and cognitive deficits in early-onset BD.

## 1. Introduction

Alternating depressive and manic or hypomanic episodes occur along with asymptomatic periods of euthymia in patients with bipolar disorder (BD), which is a mood disease marked by fluctuations in the mood state causing acute dysfunction. The prevalence of BD has exceeded 1% worldwide, and it is one of the major causes of disability in young people. Moreover, the risk of suicide in patients with BD is 20 times higher than that in the general population, and BD can lead to serious cognitive dysfunction (1, 2). The incidence of BD shows a bimodal distribution with age, with peaks at 15–24 years and 45–54 years (3). The onset of BD is before the age of 14 years in approximately 31% of patients and before the age of 20 years in approximately 59% of patients; these patients are considered to have early-onset BD (4). The presentation and clinical course of BD seem to be related to the age of onset. Patients with early-onset BD have a complex and variable condition and are prone to relapse. They tend to be more emotionally unstable, have more comorbidities, and more frequent suicide attempts and panic episodes, which eventually leads to a poor prognosis (5). Most cases of early-onset BD begin in childhood or adolescence, a period of greater biological vulnerability due to the anatomical and functional immaturity of the brain, particularly in the regions responsible for cognition, inhibition, emotion and reward (6, 7). Thus, studying the functional changes in the brains of people with early-onset BD may help improve the present understanding of the neurobiological features of BD.

Resting-state functional magnetic resonance imaging (rs-fMRI) is a neuroimaging technique that dynamically measures the blood oxygenation level-dependent (BOLD) signal in the brain, reflecting neural activity. Brain functional connectivity (FC), which is used to analyse the statistical correlation between BOLD signals from multiple brain regions at rest, has been widely used in clinical studies to observe BD features in adults (8). Anomalies in neuronal communication within certain brain networks and circuits, particularly the interactions between the default mode network (DMN) and brain regions involved in emotion processing, are the focus of research into the pathophysiological mechanisms underlying BD (9, 10). During manic and depressive episodes as well as during remission, BD patients were observed to have altered FC in the prefrontal cortex, inferior frontal cortex, cingulate cortex, thalamus, amygdala, and limbic system (11, 12). Rey et al. identified selective abnormalities in the connectivity between the amygdala and DMN regions, including reduced posterior cingulate cortex connectivity and increased medial prefrontal cortex connectivity (13). Paediatric BD patients had significantly disordered functional integration of the DMN and task-positive network/salience network, and this altered functional integration was associated with emotional and cognitive dysregulation (14). Damage across relevant networks may affect the effective cognitive and emotional activities of BD patients, who exhibit aberrant FC changes within or between certain brain networks. In fact, the human brain is never static, and the functional connections of the brain change even in a state of rest. The assumption that functional network connections remain unchanged during rs-fMRI scans does not adequately reflect the spontaneous activity of the human brain.

Dynamic functional network connectivity (dFNC) estimates time-varying FC on a temporal scale with good temporal resolution and generates high-dimensional datasets. Notably, dFNC actually captures changes in the brain’s intrinsic FC during various physiological states and may be a more sensitive marker than static FC (15–18). Long et al. found greater variability in dFNC in BD patients, which was characterised by overconnectivity of the thalamic network and the sensorimotor network (SMN) (19). Wang et al. reported reduced dFNC variability between the posterior DMN and right central executive network in a triple network model of BD (20). Large-scale dynamic and holistic brain analysis at the network level can more comprehensively describe the variation in brain FC in BD patients. The majority of recent neuroimaging studies on BD focused on static FC or dynamic FC in local networks or certain specific seed regions (20–22). The brain is a vast and sophisticated network, and anomalies in specific pathways or localised activity do not yet completely account for functional alterations at the level of the entire neural network. dFNC assists in the investigation of functional interactions between brain networks and the evaluation of their underlying network architecture without limiting the scope to a specified set of regional connections. There is a serious dearth of research on whole-brain dynamic network connection patterns in BD patients, particularly those with early-onset BD.

In this study, we compared the dFNC of 39 patients with early-onset BD with those of 22 healthy controls (HCs) matched for age, sex, and years of education. Data-driven independent component analysis (ICA) was first used to extract resting-state networks (RSNs), which exhibited spatially isolated distribution and temporally correlated functional activity in several brain regions. The time series data were then windowed, and a dFNC matrix was created using a sliding window technique. Subsequently, these matrices were clustered into different dynamic states using the k-means algorithm, and finally, a state analysis was performed to compare the dFNC and temporal metrics between the two groups. Our study aimed to determine whether dynamic patterns of organisation within the brain at the network level were altered in patients with early-onset BD and to assess the relevance of such altered network properties to clinical scores. This exploration helped to detect potentially altered functional network interactions in patients at the peak onset age and may provide some new insights to improve the present understanding of the neuropathological mechanisms of the disease.

## 2. Materials and methods

### 2.1. Participants

The study was approved by the Biomedical Ethics Committee of the First Affiliated Hospital of Nanchang University, and written informed consent was obtained from each subject or their guardians before the formal study took place. The Structured Clinical Interview-Patient Version as described in the DSM-IV-TR was used for diagnosis, and 39 patients diagnosed with early-onset BD were recruited. The severity of each patient’s emotional symptoms was assessed using the 24-item Hamilton Depression Rating Scale (HAMD-24), the Hamilton Anxiety Rating Scale (HAMA) and the Young Mania Rating Scale (YMRS) within 24 h before or after the scan. Inclusion criteria for early-onset BD patients were being 12–20 years of age and meeting the DSM-IV-TR diagnostic criteria for BD, including at least one manic, depressive, or remitting episode. At the time of scanning, all patients were either medication-naive or had been unmedicated for at least 6 months and were not receiving psychotherapy or electroconvulsive therapy. Exclusion criteria were as follows: organic brain lesions, severe physical or neurologic deficits, history of head trauma, drug or alcohol abuse, poor compliance with MRI examinations, contraindications to MRI, and substandard image quality. We also randomly recruited 22 HCs matched for age, sex, and years of education, excluding those with current or past neurological or psychiatric disorders or with any history of psychiatric disorders in first-degree relatives as determined through a Structured Clinical Interview-Nonpatient Version as described in the DSM-IV-TR. The remaining controls were subjected to the same exclusion criteria as the patient group. All subjects were right-handed according to the Edinburgh Habitual Handedness Scale criteria.

### 2.2. MRI data acquisition

All MRI data were collected in a Siemens Trio Tim 3.0 T imaging system equipped with a standard 8-channel head coil. Functional images were acquired using a single-shot gradient-echo EPI sequence with the following parameters: TR = 2000 ms, TE = 30 ms, number of slices = 30, slice thickness = 4 mm, gap = 1.2 mm, flip angle = 90°, field of view (FOV) = 200 × 200 mm^2^, matrix = 64 × 64, voxel size = 3.0 × 3.0 × 4.0 mm^3^, and number of timepoints 240 (8 min 6 s total duration). During the scan, subjects were asked to relax, close their eyes, remain still, and think of nothing in particular so that images of high quality could be obtained. The 3D T1-weighted gradient-echo sequence parameters were as follows: TR = 1900 ms, TE = 2.26 ms, number of slices = 176, slice thickness = 1 mm, gap = 0.5 mm, flip angle = 9°, FOV = 256 × 256 mm^2^, matrix = 256 × 256, and voxel size = 1.0 × 1.0 × 1.0 mm^3^.

### 2.3. Data preprocessing

The rs-fMRI data were preprocessed using GRETNA toolbox 2.0.0 in MATLAB 2014b. The preprocessing procedure included (1) discarding the first 10 time points of the images for MR signal equilibrium, (2) slice timing correction, (3) head motion correction, (4) space normalisation, registering functional data to the corresponding structural T1-weighted image and aligning the T1 images to Montreal Neurological Institute (MNI) space with resampling to a voxel size of 3 × 3 × 3 mm^3^, and (5) smoothing with an isotropic Gaussian kernel of 8 mm full width at half maximum to improve the signal-to-noise ratio.

### 2.4. Ica

The GIFT package^1^ in MATLAB 2014b was used to separate blind sources from the preprocessed fMRI data and decompose them into independent spatial components and time series. The process of ICA mainly included component estimation, data downscaling, inverse reconstruction, component selection, and postprocessing. The specific steps were as follows: (1) Component estimation: The best component number was estimated from all subjects’ fMRI data based on the minimum descriptive length (MDL) criterion, (2) Data downscaling: Principal component analysis (PCA) was used to compress and then downscale the data, and the Infomax algorithm was used for group ICA to decompose the data into independent components (ICs). The data downscaling step was repeated 100 times using the ICASSO algorithm to determine the ICs with the highest consistency and stability, (3) Inverse reconstruction: The temporal and spatial components of each subject at the individual level were reconstructed by the time–space dual regression method, and the intensity values corresponding to each voxel were Fisher-z transformed. The z-transformed data approximately obeyed a normal distribution, (4) Component selection: The Display GUI module in the GIFT software package displayed the obtained components, and ICs associated with cerebrospinal fluid, motor or vascular evoked pseudoactivation were discarded. Maximum spatial overlap and visual examination based on the Stanford functional risk standard template resulted in seven RSNs, namely, the DMN, frontoparietal network (FPN), dorsal attention network (DAN), ventral attention network (VAN), SMN, visual network (VN) and auditory network (AUN). In addition, ICA was repeated on the data from each the BD and HCs group to verify the reproducibility and reliability of the components obtained for both groups, and (5) Postprocessing: To remove scanner drift and artefacts associated with respiration, heartbeat, and movement, the selected components were subjected to detrending, despiking, low-pass filtering (0.01 ~ 0.15 Hz) and regression of the realignment parameters.

### 2.5. dFNC analysis

The dFNC matrix was calculated using a sliding window method. Previous studies found that the dynamic state changes in the brain can be effectively obtained when the window width is between 30 and 60 s (23, 24). In this study, the window width was set to a TR of 30 (60 s), and the window was slid along the time axis in steps of 1 TR. The Pearson correlation coefficients between all pairs of BOLD signals in each window were calculated to construct a series of dynamic covariance matrices. Since the covariance estimates of short time series were affected by substantial noise, we used the L1 regularisation method (number of repetitions = 10) to improve the sparsity of the dFNC matrix of each window.

The dFNC matrices of all subjects were clustered using the k-means clustering algorithm to assess the frequency and structure of recurrent dFNC patterns. In this analysis, the Manhattan city distance was used to measure the similarity between different time windows. To increase the chance of the clustering algorithm escaping local minima, we set the maximum number of iterations to 500 and the number of repetitions to 150. The elbow rule was used to determine the optimal number of clusters, *k* = 4. The dFNC matrix of all subjects was clustered into four dFNC states, which were recurrent instantaneous FC patterns across different windows and subjects. The dFNC matrix at the centre of each cluster was called the cluster centroid.

Several temporal characteristics were calculated as follows: (i) the fraction of time was defined as the ratio of the number of time windows in a state to the total number of time windows, (ii) the mean dwell time was the average time in a particular state, and (iii) the number of transitions was the number of times the subject switched from one state to another during the scan time.

### 2.6. Statistical analysis

The differences in dFNC between the BD and HCs groups were analysed directly with the Stats module of the GIFT software package using two independent samples t tests with false discovery rate (FDR) correction, and *p* < 0.05 indicated a statistically significant difference. The rest of the data were statistically analysed using SPSS 25.0 software. The age, sex, years of education, clinical neuropsychological scores, and dFNC temporal characteristics of the participants in the two groups were tested for normality separately, and measures that met the criteria for a normal distribution were expressed as the mean ± SD. The difference between the groups was compared by two independent samples t tests. The chi-square test was used to compare the proportion of each sex in the groups. The samples of dFNC temporal characteristics from the two groups were independent of each other, but none conformed to a normal distribution, so a nonparametric test (Mann–Whitney U test) was used to assess the differences between the groups, and *p* < 0.05 indicated a statistically significant difference. Finally, the relationship between dFNC temporal characteristics and clinical variables (YMRS, HAMA, and HAMD scores) was explored using Spearman’s partial correlation analysis with age, sex, and education as control variables, using Bonferroni correction, with statistically significant differences at *p* × n < 0.05.

## 3. Results

### 3.1. Demographics and clinical characteristics

After data preprocessing, 39 patients with early-onset BD and 22 HCs were finally included in this study. Demographics and clinical characteristics of the two groups are presented in Table 1; there were no differences between groups in age, sex, or years of education (all *p* > 0.05). The YMRS, HAMA and HAMD scores of early-onset BD patients were significantly higher than those of HCs (*p* < 0.0001).

**Table 1:** Demographics and clinical characteristics of all subjects.

Clinical variables (Mean ± SD)	Early-onset BD (*n* = 39)	HCs (*n* = 22)	*p* value
Age (years)	16.97 ± 2.37	17.31 ± 2.25	0.58
Sex (M/F)	19/20	11/11	0.76^*^
Handedness (R/L)	39/0	22/0	–
Educational level (years)	10.97 ± 2.37	11.32 ± 2.25	0.58
YMRS	21.56 ± 4.81	2.64 ± 2.90	<0.0001
HAMA	23.64 ± 7.04	4.18 ± 4.56	<0.0001
HAMD	32.38 ± 6.47	3.77 ± 4.81	<0.0001

^*^Represents the chi-square test *p* value for sex. M, male; F, female; R, right; L, left; SD, standard deviation.

### 3.2. Cluster analysis

**Figure 1:**
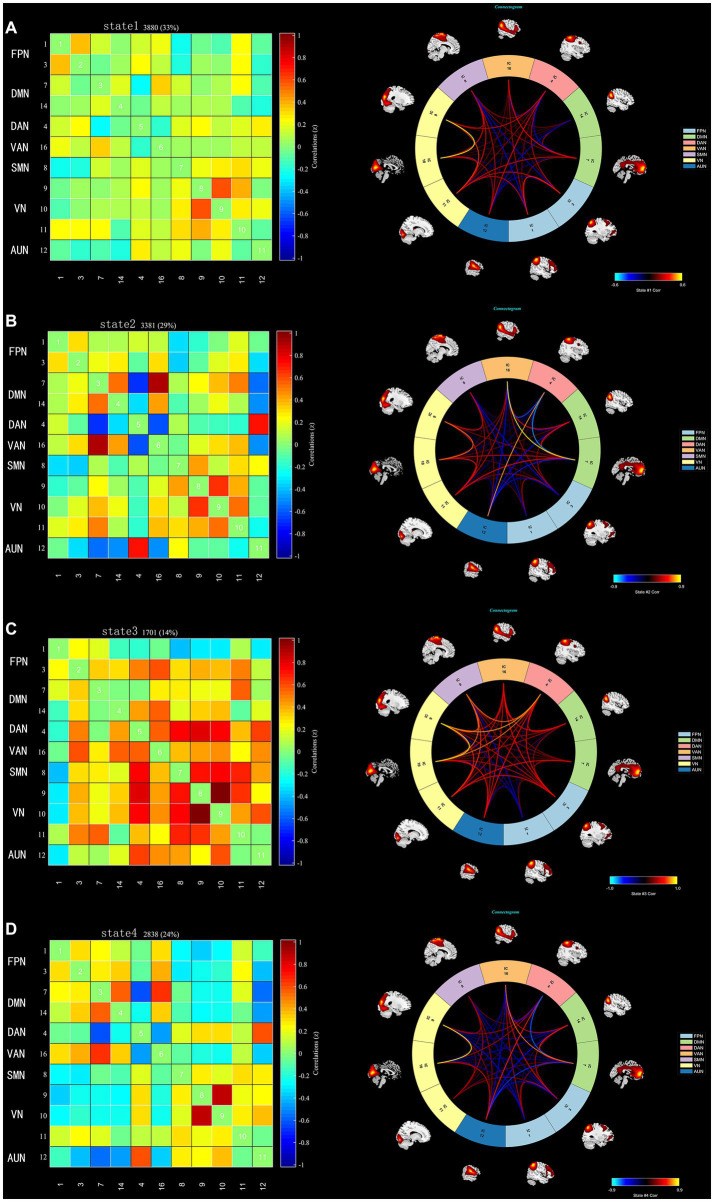
The dFNC matrices for states 1–4 and the dFNC diagrams for each state. The horizontal and vertical axes are the selected ICs and their functional networks are depicted. The colour bars indicate the z values of the dFNC. **(A)** State 1 with sparse connectivity. **(B)** State 2 with partial connectivity. **(C)** State 3 with strong connectivity, **(D)**. State 4 with modular connectivity.

**Figure 2:**
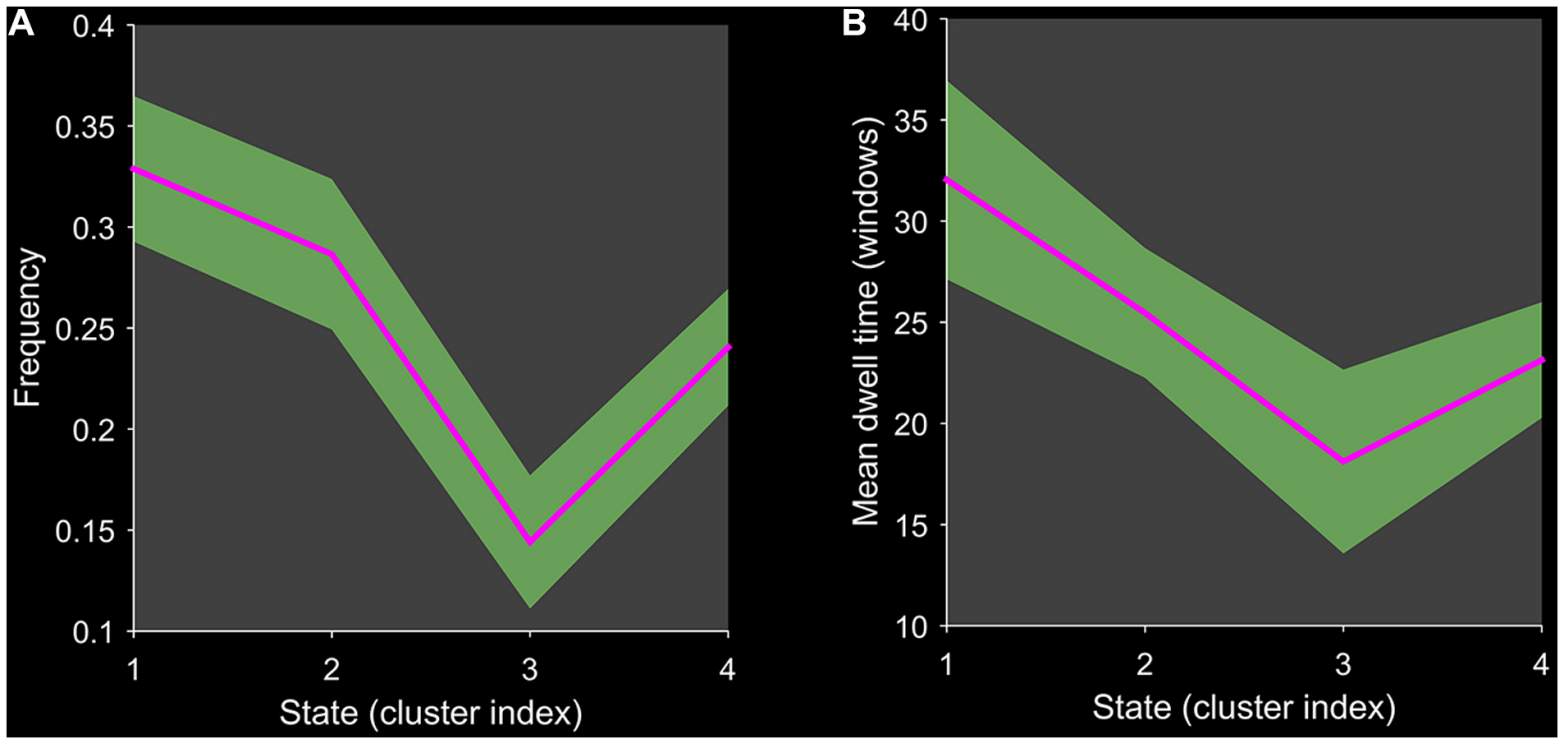
Line chart of the dFNC matrix index. Plots show **(A)** the frequency of each state and **(B)** the mean dwell time of each state.

Using the k-means clustering algorithm, we identified four recurring patterns of transient FC, i.e., dFNC states: state 1 (33% of all time windows) had sparse connectivity and exhibited general weakening of whole-brain functional network connectivity; state 2 (29% of all time windows) had partial connectivity, exhibiting increased or weakened FC within and between some networks; state 3 (14% of all time windows) showed strong connectivity, exhibiting strong positive connectivity amongst large-scale functional networks; and state 4 (24% of all time windows) was characterised by modular connectivity, which manifested as negative connectivity with obvious modularity between the FPN, DMN, SMN and the AN and AUN (Figure 1). It should be noted that not all subjects showed all four states, so the number of subjects with data demonstrating each state was not necessarily the same. The results of the dFNC matrix index of all subjects under all windows are shown in Figure 2. The data showed evidence of state 1 most frequently and had the longest mean dwell time, whilst state 3 appeared least frequently and had the shortest mean dwell time. The frequencies of transitioning from state 2 to state 1 and from state 4 to states 1 or 2 were highest amongst all subjects.

### 3.3. Comparison of dFNC temporal metrics between groups

**Figure 3:**
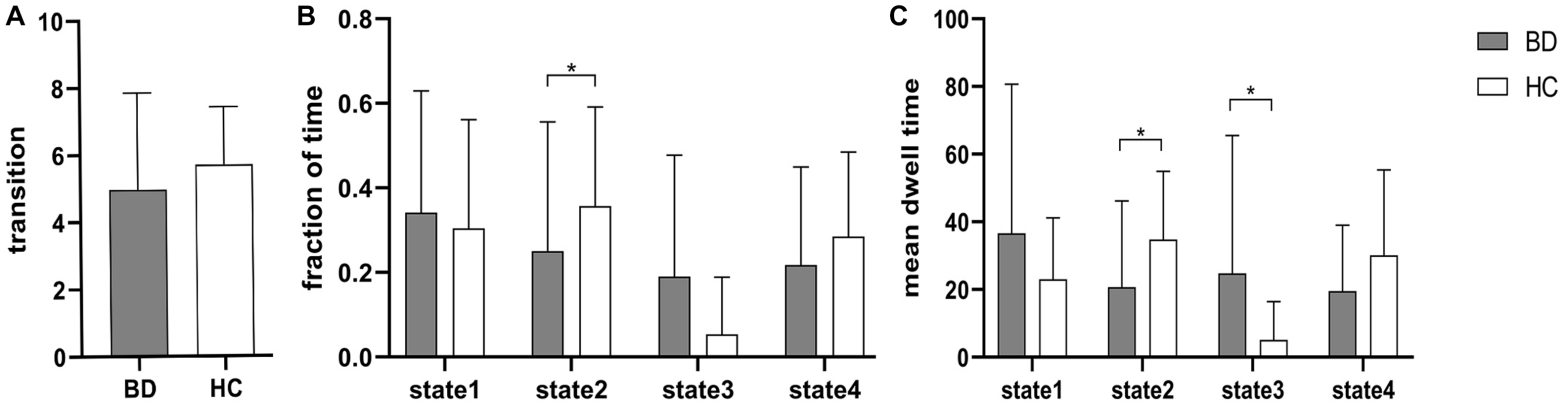
Between-group comparison of dFNC temporal metrics. Bar charts show **(A)** the number of transitions, **(B)** the fraction of time and **(C)** the mean dwell time. * above the bars indicates a significant difference between the two groups, *p* < 0.05.

Figure 3 shows that compared to HCs, BD patients had a significantly reduced fraction of time and mean dwell time in state 2 and a significantly increased mean dwell time in state 3 (Mann–Whitney U test, *p* < 0.05), but the number of transitions was not significantly different between HCs and BD patients. In the same way, the three dFNC temporal metrics (fraction of time, mean dwell time, and number of transitions) were not significantly different in state 1 and state 4.

### 3.4. Comparison of dFNC between groups

**Figure 4:**
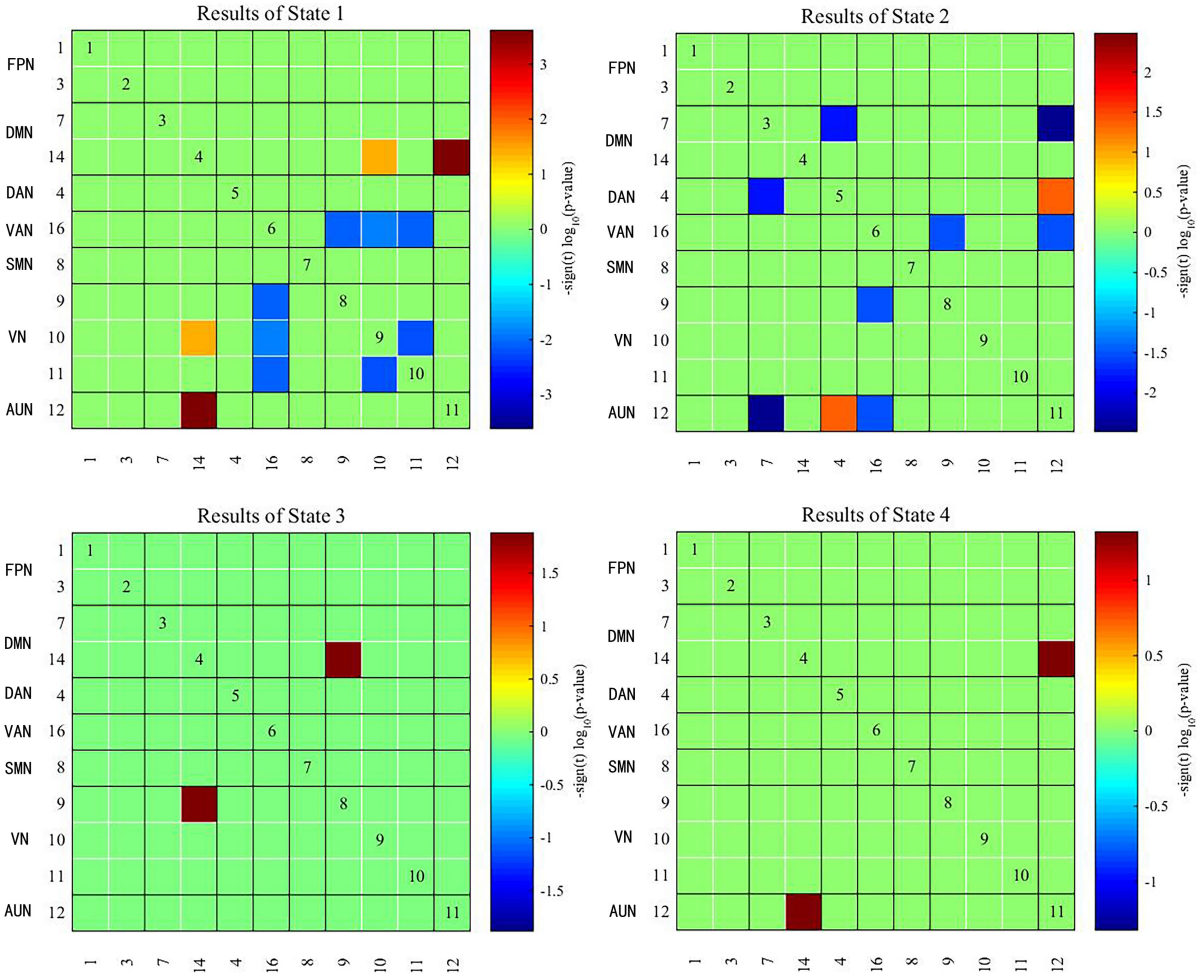
Significant differences in dFNC between early-onset BD patients and HCs. The horizontal and vertical axes represent the ICs and their functional networks. The coloured rectangles represent the dFNC between the two corresponding ICs, with warm colours indicating increased connectivity and cool colours indicating decreased connectivity.

Two independent sample t tests were used to further compare the dFNC matrix of each state between groups, and it was found that the dFNC of early-onset BD patients was significantly different in states 1–4 compared to that of HCs (FDR corrected, *p* < 0.05) (Figure 4). The specific results were as follows: The brain network connections with increased dFNC in state 1 were the DMN-VN and DMN-AUN, with the highest connectivity strength between the DMN and AUN; the brain networks with decreased dFNC were the VN and VN-VAN. The brain network connections with increased dFNC in state 2 were the DAN-AUN; the brain network connections with decreased dFNC included the DMN-DAN, DMN-AUN, VAN-VN, and VAN-AUN, with the lowest connectivity strength between the DMN and AUN. The brain network connections with increased dFNC in states 3 and 4 were the DMN-VN and DMN-AUN, respectively.

### 3.5. Correlation analysis

**Figure 5:**
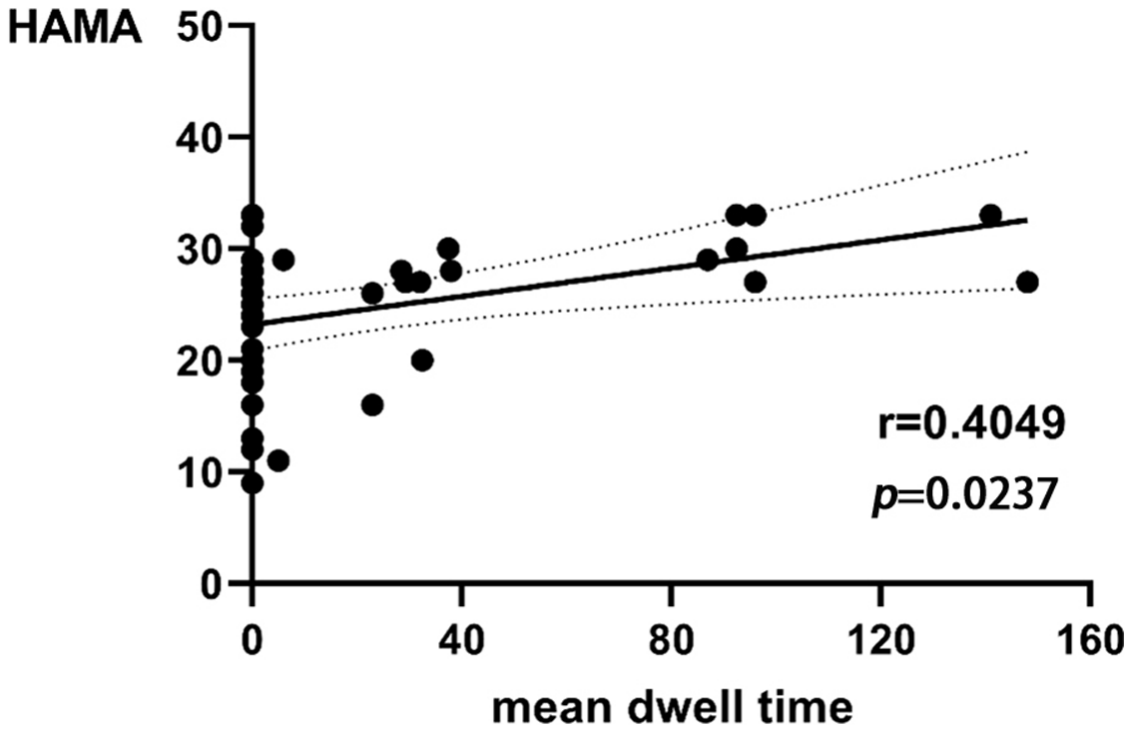
Relationship between HAMA scores and mean dwell time in state 3 of BD.

The mean dwell time in state 3 of BD was correlated with the HAMA score (*r* = 0.4049, *p* = 0.0237 × 3 > 0.05 after Bonferroni correction) in a trend manner (Figure 5). There was no significant correlation between the remaining dFNC temporal metrics and clinical neuropsychological scores.

## 4. Discussion

In this study, the differences in dFNC between patients with early-onset BD and HCs were investigated based on rs-fMRI with ICA, sliding time window and clustering analysis. The following were our key discoveries: (1) dFNC analysis showed four connected patterns: state 1 showing sparse connectivity, state 2 showing partial connectivity, state 3 showing strong connectivity, and state 4 showing modular connectivity, (2) there were significant differences between the patient group and the HC group in two temporal metrics (fraction of time and mean dwell time), and (3) in patients with early-onset BD, there was dynamic functional network reorganisation, which showed impaired coordination function between cognitive and perceptual networks.

We identified four stable and repetitive dFNC states in all subjects. The sparsely connected network in state 1 had the highest proportion of occurrence and the longest mean dwell time, possibly reflecting the average of other states that were not distinct or frequent enough to be separated and characterising the baseline activity of neurons in the resting brain (25). BD is a “disconnected disorder” in which emotional dysregulation results from the unstable neuronal dynamics of systemic circuits (26). The fraction of time and mean dwell time of state 2 were both reduced in individuals with early-onset BD compared with those of HCs, suggesting that early-onset BD patients were more likely to transition from state 2 to other states. This reduction in state 2 in patients with early-onset BD implies abnormal and more unstable information transfer within or between functional networks, ultimately suggesting abnormal local segregation and global integration of functional brain networks. State 3 was characterised by tight and strong connections. The increased mean dwell time in state 3 in early-onset BD patients suggests that there is more complex brain activity and increased abnormal information exchange and transmission within and between networks. Correlation analysis showed that the mean dwell time in state 3 in early-onset BD patients was positively correlated with the HAMA scores in a trend manner. The results must be interpreted with caution because less than half of patients with early-onset BD showed evidence of state 3 in the rs-fMRI data. Studies have shown that patients with BD and generalised anxiety disorder have functional abnormalities in brain regions, such as the frontal lobe and limbic system that are related to the severity of anxiety (27, 28). Therefore, we believe that the longer the mean dwell time is in state 3 in early-onset BD patients, the more likely their HAMA score will be elevated. However, a larger sample size is needed to verify this inference.

In recent years, the study of the functional interactions between RSNs has shown considerable promise as a potential pathway for the diagnosis of neuropsychiatric disorders (29, 30). The RSNs of the brain can be roughly divided into two categories: higher cognitive networks (the DMN, FPN, DAN, and VAN) and lower sensory and perceptual networks (the SMN, VN, and AUN). Intranetwork and internetwork connectivity reflect the segregation and integration of brain information, respectively (31). Previous studies have shown that BD is associated with a wide range of FC deficits that are thought to be caused by abnormalities in the integration and segregation of brain networks (32). BD patients have more abnormal internetwork connectivity than intranetwork connectivity, suggesting the importance of cross-regional cooperation between brain regions in emotion regulation and cognitive function (33), which is similar to our findings. In our study, patients with early-onset BD showed reduced connectivity amongst many brain networks in state 2 compared to that of controls, especially between the DMN and AUN. The DMN is considered a key component of the functional structure of the brain, involving self-referential and reflective activities that are preferentially activated in resting states and inhibited in a wide range of cognitive tasks. In addition, the DMN acts as a “cohesion connector,” exhibiting high network cohesion and high internetwork integration and integrating information from primary functional and cognitive networks to support a wide range of brain functions (7, 34). It has been shown that the functional separation and integration of the DMN in BD patients is inefficient, which may imply impaired local processing and long-distance information transfer in the DMN (9, 35, 36). The AUN includes the primary auditory cortex and the secondary auditory cortex. The primary auditory cortex mainly encodes the properties of the auditory stimulus, whilst the secondary auditory cortex integrates and connects this information to produce a specific percept (37). The decreased FC between the DMN and AUN indicated that external perception ability and self-consciousness integration were impaired in early-onset BD patients. The DAN is primarily responsible for attentional orienting and visual and spatial perception, and it has the function of allocating of cognitive resources in response to stimuli (38, 39). The interaction between the internal cognitive processing network (DMN) and the external directed cognitive network (DAN) reflects the switch between the intrinsic and extrinsic focus of attention (40). The reduced FC between the two networks in this study may explain the abnormal control of attention in early-onset BD patients. In addition, many abnormalities in the FC of other brain regions within the cognitive network, within the perceptual network, and between the two were also observed in patients with early-onset BD, which further demonstrated the existence of abnormal local segregation and global integration of functional brain networks in early-onset BD patients.

In the present study, the brain networks with increased dFNC in early-onset BD patients in state 1 were the DMN-VN and DMN-AUN. Notably, increased dFNC of the DMN-VN and DMN-AUN also occurred in state 3 and state 4. The dFNC enhancement between the DMN and VN and between the DMN and AUN in multiple states may be characteristic of brain network damage in early-onset BD patients. Previous studies by our group identified a possible developmental pattern of hyperconnectivity supporting switching between higher cognitive networks and primary perceptual networks in patients with early-onset BD, which generally occurs during childhood and adolescence. The VN and AUN are involved in the perceptual processing of information exchange with the external environment. The higher dFNC between these networks compared with the normal state may be related to patients’ greater sensitivity to external visual or auditory stimuli and emotions (41). Different patterns of visual attention deficits in BD patients were associated with different external emotional stimuli, suggesting the interactive influence of cognitive and emotional functions (42). Biological activities, such as head or body movements, facial expressions, and auditory stimuli stimulate the brain to engage in a wide range of cognitive activities (43, 44), which may explain the cognitive deficits seen in BD patients. Thus, the abnormalities of neural circuits amongst the DMN, DAN, VN and AUN in early-onset BD patients may be responsible for the abnormal reception of external stimuli, psychological activity and cognitive performance compared with those of HCs. Abnormal dynamic brain network reorganisation may constitute a potential imaging biomarker for early-onset BD. Furthermore, we inferred that the enhanced integration of the DMN may represent an adaptive response to the diminished integration of the DMN observed in state 2. This interpretation is speculative and must be confirmed by further studies.

Although audiovisual dysfunction is not a typical clinical symptom of BD, studies have shown that neurophysiological deficits and poorer task performance in adolescent BD imply that visual and auditory pathways do not transfer sensory data well to the audiovisual cortex and that the frontal cortex does not integrate incoming signals well into unified and coherent perceptual actions (45). Our results showed abnormal connectivity between the VN and higher cognitive networks (i.e., the DMN, DAN, and VAN), the AUN and higher cognitive networks, and within the VN across multiple states in early-onset BD. This connectivity reflects the disrupted dynamic reconfiguration of sensory and perceptual systems and a failure of dynamic integration of higher-order processes, which may underlie the perceptual and cognitive deficits in early-onset BD. This study also further validated our group’s previous findings that audiovisual integration relies on a brain network mechanism of feedback information flow between higher-order networks and the underlying perceptual cortex.

In this study, we found abnormal dynamic properties of whole-brain functional network connectivity in early-onset BD by measuring dFNC; these changes may imply abnormal separation and integration processes in the brain. Patients with early-onset BD had impaired coordination between cognitive and perceptual networks, which specifically manifested as impaired coordination between internal introspection and external environmental detection, as well as perceptual dysfunction. In addition, the specific dFNC temporal metrics were altered in early-onset BD patients, which may provide a new imaging basis for clinical symptom prediction and assessment in these patients.

Our study had several limitations. First, the present study had a relatively small sample size, and we did not conduct a grouping study that considered the different subtypes of early-onset BD. There may be heterogeneity amongst different subtypes. The sample size should be expanded for a more precise grouping study in the future. In addition, correlation analysis required a larger sample size for validation. Second, the study protocol did not include collection of relevant cognitive test scores and behavioural measures, and these incomplete scales limited our interpretation of cognitive-related networks. Third, the choice of sliding window size may influence the assessment of dynamic brain connections. Future analyses should be performed using different window widths to verify the reliability and reproducibility of the results. Finally, our study was a cross-sectional study lacking longitudinal comparisons, so the findings may have certain limitations.

## Data availability statement

The raw data supporting the conclusions of this article will be made available by the authors, without undue reservation.

## Ethics statement

The studies involving human participants were reviewed and approved by Biomedical Ethics Committee of the First Affiliated Hospital of Nanchang University. Written informed consent to participate in this study was provided by the participants' legal guardian/next of kin.

## Author contributions

ZH: writing - original draft, data curation, software, proof Checking. CZ: investigation, modified the language, revision, proof Checking. LH: supervision and writing - review and editing. All authors contributed to the article and approved the submitted version.

## Funding

This study was funded by the National Natural Science Foundation of China (Grant/Award Number: “81460329”) and the Natural Science Foundation of Jiangxi Province (Grant/Award Numbers: “20192ACBL20039,” and “202310454/202310456”).

## Conflict of interest

The authors declare that the research was conducted in the absence of any commercial or financial relationships that could be construed as a potential conflict of interest.

## Publisher’s note

All claims expressed in this article are solely those of the authors and do not necessarily represent those of their affiliated organizations, or those of the publisher, the editors and the reviewers. Any product that may be evaluated in this article, or claim that may be made by its manufacturer, is not guaranteed or endorsed by the publisher.

## Footnotes

^1^ http://icatb.sourceforge.net/
